# AI in Disaster Medicine: Scoping Review of Methods, Validation, and System Integration

**DOI:** 10.2196/90848

**Published:** 2026-08-04

**Authors:** Ruben Peralta, Ali Msheik, Zeinab Al Mokdad, Yavuz Yigit, Hassan Al-Thani, Ghaya Al Rumaihi, Ghanem Al-Sulaiti, Peter Cameron

**Affiliations:** 1 Trauma Surgery, Hamad Medical Corporation, Doha, Baladīyat ad Dawḩah, Qatar; 2 Department of Surgery, Universidad Nacional Pedro Henriquez Urena, Santo Domingo, 10100, Dominican Republic; 3 Neurological Surgery, Neuroscience Insitute, Hamad Medical Corporation, P.O. Box: 3050, Doha, Baladīyat ad Dawḩah, Qatar, 974 72157731; 4 Department is Bioethics and Medical Ethics, Faculty of Medical Sciences, Lebanese University, Beirut, Beyrouth, Lebanon; 5 Corporate Department of Emergency Medicine, Hamad Medical Corporation, Doha, Baladīyat ad Dawḩah, Qatar; 6 Emergency Medicine, Centre for Neuroscience and Trauma, Blizard Institute, Queen Mary University of London, London, United Kingdom; 7 College of Medicine, Qatar University, Doha, Baladīyat ad Dawḩah, Qatar

**Keywords:** disaster medicine, artificial intelligence, AI, emergency response, decision-support systems, mass casualty incidents

## Abstract

**Background:**

AI is increasingly proposed as a tool to enhance disaster medicine through improved situational awareness, decision support, and resource coordination. However, the extent to which current research has progressed beyond methodological development toward integrated, operationally validated systems remains unclear.

**Objective:**

This review aimed to systematically map the scope, methods, validation strategies, and system integration of AI applications in disaster medicine and emergency health care systems.

**Methods:**

This scoping review was conducted in accordance with PRISMA-ScR (Preferred Reporting Items for Systematic Reviews and Meta-Analyses Extension for Scoping Reviews) guidelines. The PubMed (MEDLINE), Scopus, IEEE Xplore, and Google Scholar databases were searched from inception to January 31, 2026. Studies describing AI applications in disaster medicine, emergency response, mass casualty care, or public health emergencies were eligible. Data were charted across the emergency domain, scenario type, AI function, study design, and validation level.

**Results:**

A total of 168 studies were included. Research activity was concentrated in the disaster response and rescue, and public health and pandemics domains, which together accounted for 64 (38.1%) studies. Most studies involved algorithm or model development (43/168, 25.6%) or system or tool development (33/168, 19.6%), whereas applied and observational studies were less common (15/168, 8.9%). Validation was predominantly internal or simulation-based; external validation was reported in 13 (7.7%) studies, and prospective real-world validation was reported in 2 (1.2%) studies. Human-centered, smart city, and mental health domains were consistently underrepresented.

**Conclusions:**

AI research in disaster medicine is expanding rapidly but remains fragmented and is at an early stage of translational maturity. Future progress will depend on system-level integration, rigorous real-world validation, and alignment with operational emergency workflows.

## Introduction

### Background

Disasters pose a persistent and evolving threat to health care systems worldwide, including natural hazards, technological incidents, infectious disease outbreaks, and mass casualty events. Disaster medicine (DM) is a multidisciplinary field concerned with the health sector’s roles across mitigation, preparedness, response, recovery, and resilience during disasters while maintaining continuity of essential health services [1,2].

AI, including machine learning, deep learning, natural language processing, optimization, and rule-based decision support, has emerged as a promising set of methods for addressing these challenges. In this review, AI was operationally defined to include data-driven prediction, classification, detection, optimization, and decision-support approaches applied to disaster-related or emergency health contexts. AI-driven systems have shown potential to provide early warning, surge prediction, triage support, resource optimization, and real-time decision support across health care and emergency settings [3].

Despite growing interest, the application of AI in DM remains fragmented. Existing studies vary widely in scope, methodology, and implementation context, and many rely on retrospective datasets, simulations, or single-system analyses [2-4]. Prospective, real-time, and health care system–integrated applications appear to be limited. A comprehensive mapping of the current evidence is therefore needed to identify research gaps, implementation barriers, and priorities for future research [4].

Scoping reviews are well suited to this purpose, as they enable the systematic mapping of heterogeneous evidence, the clarification of key concepts, and the identification of gaps in knowledge without restricting inclusion to narrowly defined study designs. This approach is particularly appropriate at the intersection of DM and AI, where innovation often precedes standardized evaluation.

### Objectives

The objective of this scoping review was to map and characterize the existing literature on the application of AI in DM and disaster management, including the AI methods used; the disaster management cycle contexts in which they were applied; the data sources and health care system settings involved; the study designs and validation approaches used; the geographic distribution of the evidence; and the methodological, operational, and ethical challenges reported.

## Methods

### Overview

This scoping review was conducted in accordance with the PRISMA-ScR (Preferred Reporting Items for Systematic Reviews and Meta-Analyses Extension for Scoping Reviews) guidelines [5]. A completed PRISMA-ScR checklist is provided in Checklist 1.

### Ethical Considerations

Ethics approval was not required because this study was a scoping review of published literature. All data analyzed in this study are available in the cited literature.

### Eligibility Criteria

Studies were selected according to the criteria detailed in Table 1.

**Table 1.** Inclusion and exclusion criteria.

Criteria	Inclusion criteria	Exclusion criteria
Technology or intervention	AI, machine learning, deep learning, predictive analytics, or algorithm-based decision support	AI applications unrelated to disaster or emergency health care contexts
Disaster or emergency context	Disaster medicine, emergency preparedness, or response, mass casualty incidents, or public health emergencies	Non–health care disaster domains only
Setting or system	Health care systems, emergency medical services, hospital systems, or public health operations	Military-only or cybersecurity-only applications without health care relevance
Study design	Any empirical or methodological study design	Editorials, commentaries, opinion pieces, or narrative-only reports without methodological content
Language	English-language publications	Non–English-language publications

### Information Sources and Search Strategy

A comprehensive search strategy was developed for PubMed (MEDLINE), Scopus, IEEE Xplore, and Google Scholar. All sources were searched from inception to January 31, 2026. PubMed (MEDLINE), Scopus, and IEEE Xplore were selected to capture biomedical, multidisciplinary, and engineering literature relevant to AI applications in DM. Google Scholar was used as a supplementary search source because of its broad indexing of scholarly and technical material, including conference proceedings and other nontraditional records. Because Google Scholar does not support the same controlled vocabulary or reproducible advanced syntax as bibliographic databases, targeted phrase-based searches were conducted using combinations of terms related to AI, DM, emergency response, mass casualty incidents, public health emergencies, and health care system applications. The most relevant records retrieved for each query were screened, deduplicated against database records, and assessed using the same eligibility criteria as all other sources. The complete source-specific search strategies, including the Google Scholar queries and screening approach, are provided in Multimedia Appendix 1.

### Study Selection

All identified records were imported into a reference management system, and duplicate records were removed. Titles and abstracts were screened independently by at least 2 reviewers for relevance. Full texts of potentially eligible studies were then assessed against the inclusion and exclusion criteria by at least 2 reviewers. Discrepancies were resolved through discussion and consensus.

### Data Charting and Synthesis of the Results

A standardized data-charting form and coding framework were developed and pilot-tested before full extraction. The coding categories were selected a priori to align with the review objectives and scoping review methodology, which emphasizes mapping the breadth, characteristics, and gaps of heterogeneous evidence rather than estimating pooled effects. The coding framework captured the main dimensions needed to characterize AI applications in DM: emergency domain, scenario type, AI function, study design, validation approach, geographic origin, and descriptive characteristics relevant to implementation and system integration. These variables were chosen because they reflect the key translational questions of the review: where AI is applied, what functions it supports, how systems are evaluated, and how close the evidence is to operational implementation. Data were charted by at least 2 reviewers using these predefined categories. Disagreements were resolved through discussion and consensus. Results were synthesized descriptively across these thematic domains. Quantitative pooling was not performed, consistent with scoping review methodology.

## Results

### Overview

**Figure 1.**
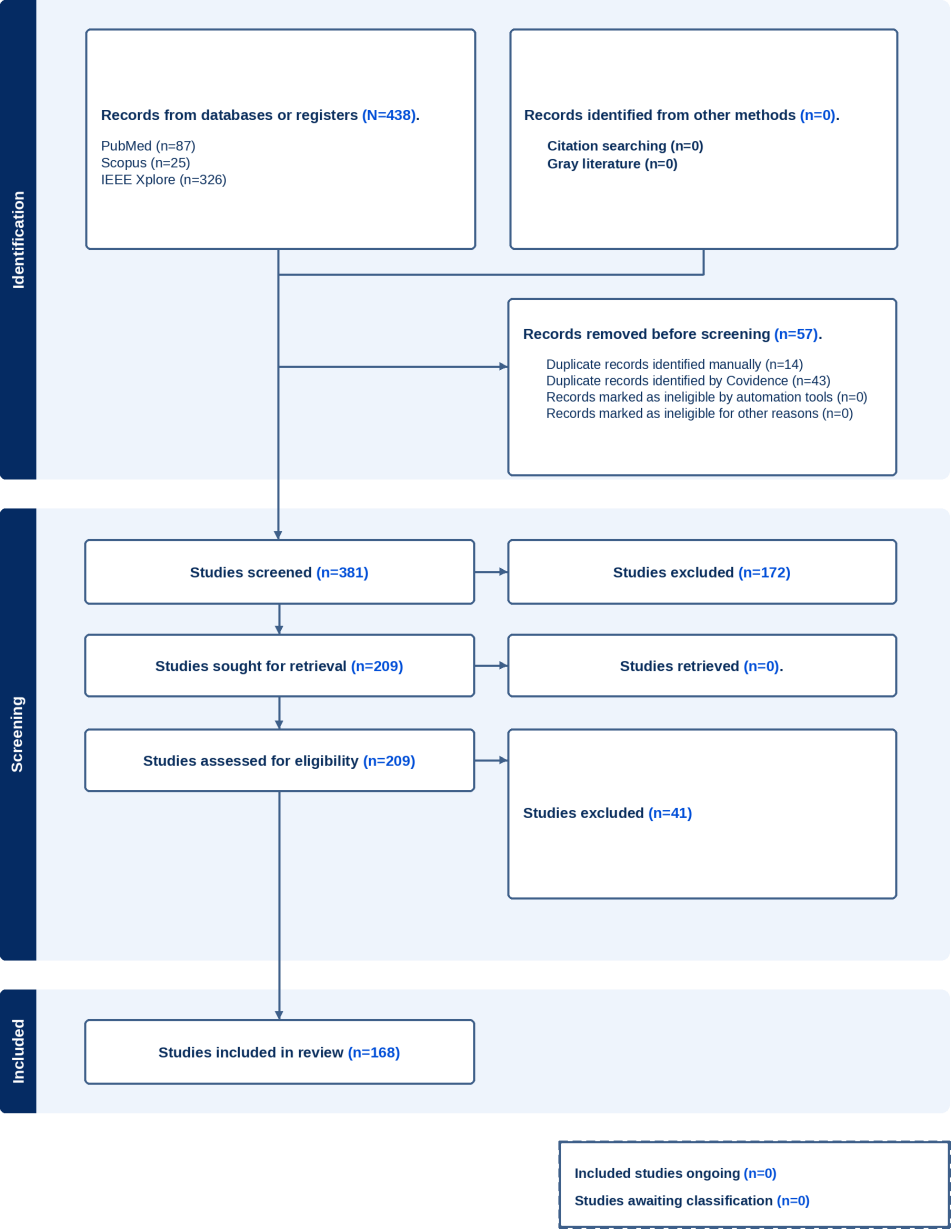
PRISMA-ScR (Preferred Reporting Items for Systematic Reviews and Meta-Analyses Extension for Scoping Reviews) flow diagram of study selection.

A total of 438 records were identified through database searching. After the removal of 57 duplicate records, 381 unique records remained for screening. Following title and abstract screening, 209 records underwent full-text review. Of these, 168 studies met the inclusion criteria and were included in the final synthesis (Figure 1). A detailed study characteristics table is provided in Multimedia Appendix 2.

Google Scholar records were screened as a supplementary source and deduplicated against records retrieved from the PubMed (MEDLINE), Scopus, and IEEE Xplore databases. After screening and deduplication, Google Scholar did not contribute additional unique studies to the final included set. This finding refers only to the source through which records were identified and does not indicate that gray or technical literature was absent from the review. Several included records were conference proceedings or technical studies, particularly from IEEE Xplore, and were therefore classified as part of the broader gray or technical literature contribution to the evidence base.

**Figure 2.**
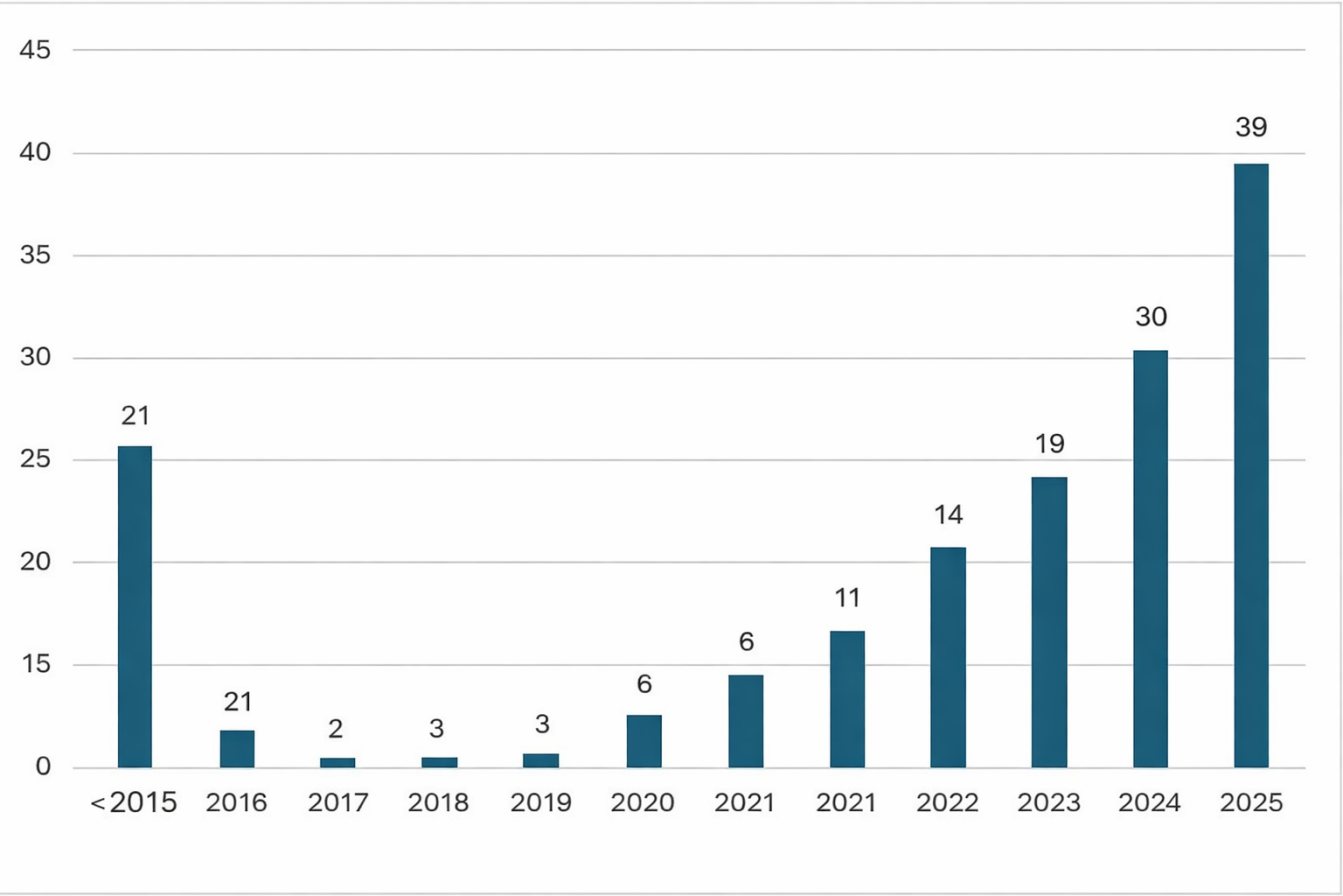
Distribution of included studies by publication year.

The temporal distribution of included studies showed an increase in research output in recent years, with most studies published after 2020. Publication activity was highest in 2024 (30/168, 17.9%) and 2025 (39/168, 23.2%), which together accounted for 69 (41.1%) studies in the included evidence base. Fewer studies were published in earlier years, with only sporadic contributions before 2015 (Figure 2).

**Figure 3.**
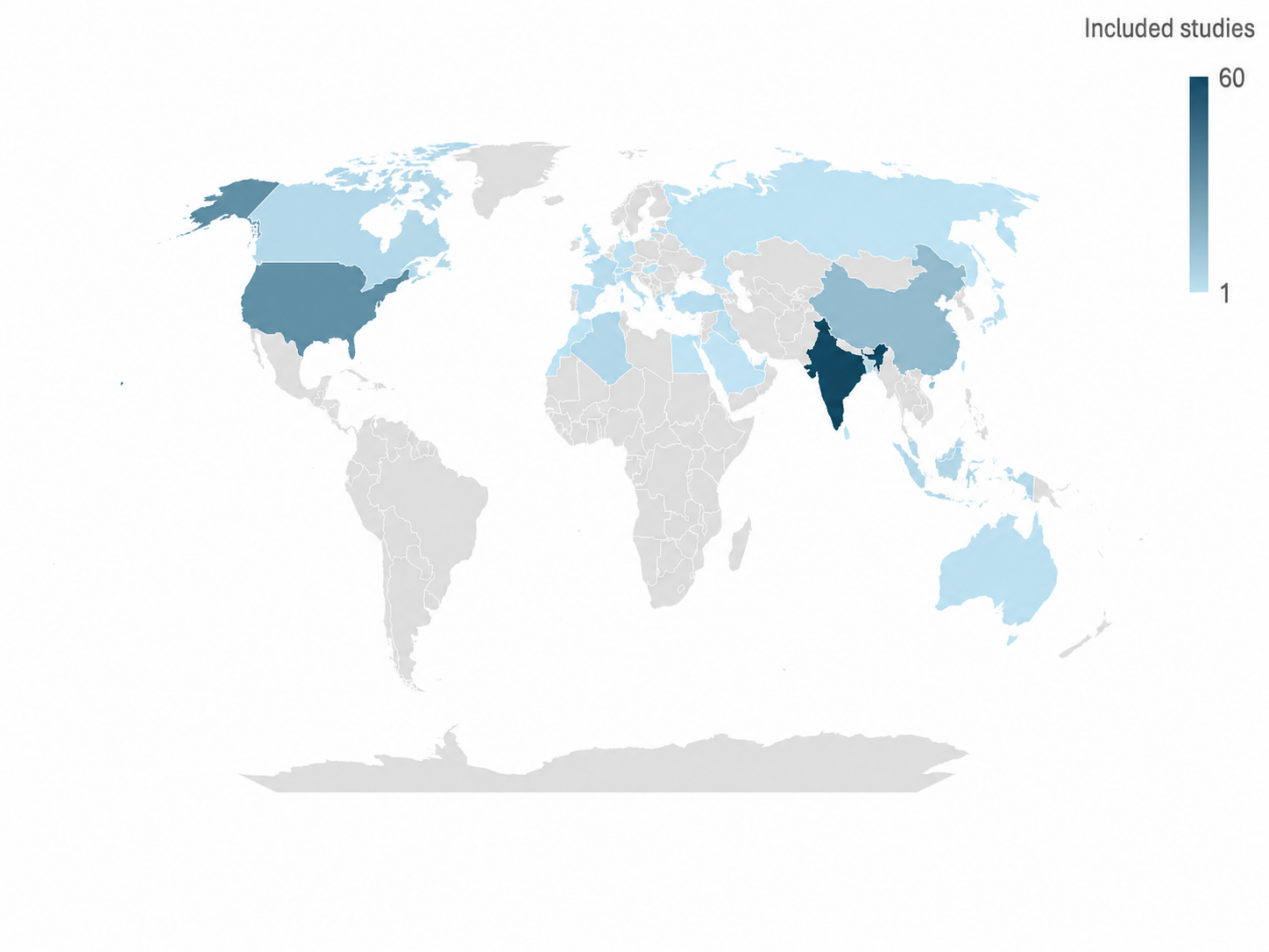
Geographic distribution of the included studies by country or region. Map data are provided by the Australian Bureau of Statistics, GeoNames, Microsoft, Navinfo, Open Places, OpenStreetMap, Overture Maps Foundation, TomTom, and Zenrin and are powered by Bing.

Geographically, research output was unevenly distributed, with the highest number of studies originating from South Asia, driven largely by contributions from India, followed by North America, East Asia, and Europe. In contrast, Africa, South America, and Central Asia were minimally represented, indicating substantial geographic disparities in the evidence base (Figure 3).

Across all analytical dimensions, the evidence base demonstrated a marked concentration within a limited number of categories, and publication types included both journal articles and conference proceedings or other technical studies (Table 2). Learning-based AI approaches dominated, with machine learning, deep learning, and hybrid systems forming the methodological core, whereas symbolic, multiagent, simulation-based, and vision-focused approaches were comparatively infrequent. Publication sources were also concentrated in journal articles and conference proceedings, with the latter representing a substantial proportion of the included evidence.

**Table 2.** Distribution of included studies across analytical domains and subcategories (N=168)^a^.

Domains and categories	Studies, n (%)		References
Study design			
Algorithm or model development	43 (25.6)		[6-8]
System or tool development	33 (19.6)		[9-11]
Experimental or evaluation study	26 (15.5)		[12-14]
Simulation-based study	17 (10.1)		[15-17]
Applied or operational system study	15 (8.9)		[13,18,19]
Observational or case-based study	14 (8.3)		[18-20]
Framework or conceptual study	12 (7.1)		[21-23]
Review, survey, or meta-study	5 (3)		[24-26]
Review or evidence synthesis	4 (2.4)		[3,27]
Validation type			
Internal experimental validation	57 (33.9)		[28-30]
No or not reported validation	34 (20.2)		[21,31,32]
Simulation-based validation	31 (18.5)		[12,15,16]
Retrospective or dataset-based validation	19 (11.3)		[33-35]
External or real-case validation	13 (7.7)		[13,18,19]
Conceptual or prototype-level validation	8 (4.8)		[22,36,37]
Prospective or real-world validation	2 (1.2)		[18,19]
User or human-in-the-loop validation	2 (1.2)		[38,39]
Pilot or feasibility validation	1 (0.6)		[18]
AI type			
Machine learning	45 (26.8)		[30,40,41]
Deep learning	35 (20.8)		[42-44]
Hybrid AI systems	25 (14.9)		[28,45,46]
Optimization and metaheuristics	21 (12.5)		[47-49]
Natural language processing	10 (6)		[33,50,51]
Intelligent decision-support systems	9 (5.4)		[9,22,52]
Reinforcement learning	8 (4.8)		[17,53,54]
Knowledge-based or rule-based AI	6 (3.6)		[10,22,55]
Multiagent systems	3 (1.8)		[56-58]
Computer vision	3 (1.8)		[59-61]
Ensemble machine learning	1 (0.6)		[28]
Multiagent and simulation AI	1 (0.6)		[17]
Emergency domain			
Public health and pandemics	37 (22)		[34,35,43,62-79]
Disaster response and rescue	27 (16.1)		[13,80-94]
Disaster management and preparedness	27 (16.1)		[10,23,25,95-107]
Emergency and critical care	17 (10.1)		[3,28,108-117]
Emergency medical services and prehospital care	12 (7.1)		[118-124]
Critical infrastructure and resilience	10 (6)		[53,125-131]
Mass casualty incidents	10 (6)		[6,8,52]
Disaster risk reduction and recovery	6 (3.6)		[23,132-139]
Public safety and crisis management	6 (3.6)		[44,140-145]
Disaster medicine	3 (1.8)		[1,146-148]
Health care systems	3 (1.8)		[46,149-151]
Crisis communication and information systems	3 (1.8)		[50,152,153]
Disaster monitoring and assessment	2 (1.2)		[154-156]
Emergency medical education and training	2 (1.2)		[38,157]
Smart city resilience	2 (1.2)		[158-161]
Smart city crisis management	1 (0.6)		[162]
Disaster mental health	1 (0.6)		[163,164]
Prehospital and mass gathering medicine	1 (0.6)		[118,165]
Scenario type			
Disaster response and rescue operations	51 (30.4)		[13,87,93]
Disaster preparedness, planning, and training	35 (20.8)		[10,23,38]
Pandemic and infectious disease events	26 (15.5)		[34,43,166]
Climate, weather, and natural hazards	23 (13.7)		[41,42,167]
Public safety, mobility, and urban emergencies	13 (7.7)		[36,144,159]
Emergency medical and health care	9 (5.4)		[3,28,113]
Disaster recovery and postdisaster systems	6 (3.6)		[20,136,138]
Mass casualty and high-impact events	5 (3)		[6-8]
AI function			
Prediction and forecasting	31 (18.4)		[6,35,42]
Detection and early warning	30 (17.8)		[29,168,169]
Decision support and clinical support	25 (14.9)		[9,28,52]
Monitoring and surveillance	20 (11.9)		[59,170,171]
Situational awareness and intelligence	18 (10.7)		[17,44,152]
Coordination, routing, and logistics	17 (10.1)		[47,48,172]
Resource allocation and optimization	16 (9.5)		[8,30,49]
Training, preparedness, and capacity building	6 (3.6)		[38,157,173]
Simulation, planning, and evaluation	4 (2.4)		[12,15,16]
Communication, awareness, and public engagement	2 (1.2)		[50,153]

a Conference proceedings and technical studies were classified by publication type and should not be interpreted as records uniquely identified through Google Scholar.

Research activity across emergency domains was heavily clustered within public health and pandemics, disaster response and rescue, and disaster management and preparedness, with minimal representation in smart city, mental health, and mass gathering medicine–specific domains. Similarly, scenario types were dominated by disaster response, preparedness, pandemic, and climate-related events, with comparatively limited attention given to recovery-focused or mass casualty event–specific scenarios.

In terms of AI function, most studies addressed prediction, detection, decision support, and monitoring, whereas coordination, logistics, training, simulation, and public communication functions were less frequently explored (Table 2). Study designs were predominantly algorithmic or system development oriented, with fewer applied, observational, or synthesis-based studies. Validation strategies were largely confined to internal experimental or simulation-based approaches, whereas real-world, prospective, and human-in-the-loop validation remained rare, underscoring persistent gaps in translational maturity.

### Scenario Type Across Emergency Domains

As shown in Table 2, scenario types broadly aligned with their corresponding operational domains. Disaster response and rescue operations constituted the largest scenario category, with 51 (30.4%) studies, predominantly mapping to the disaster response and rescue domain, with additional contributions from emergency and critical care and emergency medical services and prehospital care, reflecting the multidisciplinary nature of real-time disaster operations. Disaster preparedness, planning, and training accounted for 35 (20.8%) studies and was commonly represented within emergency and critical care, public health and pandemics, and mass casualty incidents, indicating a health care system–centered preparedness focus. Pandemic and infectious disease events comprised 26 (15.5%) studies and were concentrated within the public health and pandemics domain, with comparatively limited integration into the emergency response or critical care domains. Climate, weather, and natural hazard scenarios accounted for 23 (13.7%) studies and were primarily aligned with disaster management and preparedness and critical infrastructure and resilience, emphasizing anticipatory and resilience-oriented approaches. In contrast, disaster mental health, smart city crisis management, and smart city resilience were sparsely represented across all scenarios, highlighting persistent gaps between emerging conceptual priorities and empirical implementation.

### Methodological Distribution Across Emergency Domains

The distribution of study designs across emergency domains indicates a strong methodological skew toward algorithmic and system-oriented research. Algorithm and model development constituted the largest category overall, with 43 (25.6%) studies. This pattern was especially evident in disaster management and preparedness and public health and pandemics, reflecting a focus on predictive modeling, decision algorithms, and optimization frameworks rather than implementation-ready systems. Disaster response and rescue appeared methodologically diverse, combining algorithmic work, experimental evaluations, and system or tool development, suggesting a comparatively higher level of translational intent than that of other domains. In contrast, emergency and critical care showed a more balanced mix of algorithm development, applied system studies, and observational or case-based research, consistent with the availability of clinical data and real-world constraints. Review-based evidence synthesis remained limited across all domains, and observational or real-world case studies were underrepresented, particularly in infrastructure-focused and smart city domains. Overall, Table 2 highlights a field largely driven by algorithmic and system-oriented research, with comparatively fewer applied or observational studies.

### Validation Level Across Emergency Domains

Analysis of validation approaches across emergency domains revealed a predominance of early-stage and internally validated research, with limited progression toward real-world deployment. Internal experimental validation was the most common approach, reported in 57 (33.9%) studies, particularly within disaster management and preparedness, disaster response and rescue, emergency and critical care, and public health and pandemics. In contrast, external or real-case validation remained relatively uncommon, being reported in 13 (7.7%) studies, primarily within disaster response and rescue and public health and pandemics. Simulation-based validation accounted for 31 (18.5%) studies, especially in critical infrastructure and resilience, mass casualty incidents, and emergency medical services and prehospital care, underscoring the reliance on modeled or hypothetical scenarios rather than operational testing. No or unreported validation was observed in 34 (20.2%) studies, spanning nearly all major domains. Domains such as smart city crisis management, smart city resilience, and disaster mental health exhibited particularly low validation maturity, with minimal empirical testing and no prospective or real-world validation. Collectively, these findings suggest limited progression from internal and simulation-based validation toward external or real-world testing across emergency domains.

### Integrated Cross-Domain Synthesis of Gaps and Concentration

Integrating findings across emergency scenarios, methodological approaches, and validation levels reveals a highly uneven research landscape characterized by strong thematic concentration and limited translational maturity. Research activity was heavily clustered within disaster response and rescue (27/168, 16.1%) and public health and pandemics (37/168, 22%), together accounting for more than one-third of the evidence base. Although these domains spanned the widest range of scenario types and methodological designs, this apparent diversity was driven primarily by algorithm and model development (43/168, 25.6%) and system or tool development (33/168, 19.6%), with comparatively few studies advancing to applied operational evaluation. Across all domains, only 13 (7.7%) studies achieved external or real-case validation, and only 2 (1.2%) studies reported prospective real-world validation, underscoring limited progression toward deployment-ready solutions. In contrast, domains such as disaster mental health (1/168, 0.6%), smart city crisis management (1/168, 0.6%), and smart city resilience (2/168, 1.2%) remained markedly underrepresented across scenario types, study designs, and validation levels, suggesting that these areas remain largely conceptual rather than empirically tested. Critical infrastructure and resilience (10/168, 6%) and mass casualty incidents (10/168, 6%) demonstrated moderate scenario coverage but relied predominantly on simulation-based or retrospective dataset-based validation, limiting confidence in their real-world applicability. Notably, 34 (20.2%) studies reported no validation or did not specify a validation approach. Overall, although methodological sophistication in emergency and disaster research is advancing, the dominance of internally validated and simulation-driven studies highlights a persistent gap between technical innovation and mature, field-tested, system-level implementation.

## Discussion

This scoping review found that the literature on AI in DM is expanding rapidly but remains concentrated in a small number of emergency domains, dominated by algorithm development and system development studies, and characterized by limited external or prospective real-world validation. Research activity was particularly concentrated in public health and pandemics and disaster response and rescue, whereas human-centered, smart city, and mental health domains were sparsely represented (Table 2) [13,34,35,43,87,93,163].

### Concentration of Research Activity and System-Level Fragmentation

The strong concentration of studies within the disaster response and rescue and public health and pandemics domains likely reflects global priorities shaped by climate-related disasters, mass casualty events, and infectious disease outbreaks. However, the cross-domain analysis suggests that much of this activity remains compartmentalized. Many studies address isolated components of the emergency continuum, such as detection, prediction, or triage, rather than integrated support across prehospital, hospital, and public health decision-making. This pattern is consistent with representative studies of triage support, surge prediction, and pandemic analytics [3,6,34] and suggests limited end-to-end operational integration within the current evidence base, particularly across prehospital, hospital, and public health interfaces [13,118,120,122].

### Methodological Emphasis Versus Operational Readiness

The predominance of algorithm and model development, often evaluated through internal or simulation-based validation, suggests a persistent mismatch between technical development and operational readiness. Although such studies are important for methodological innovation, their dominance indicates that comparatively few systems have progressed to applied evaluation within real emergency workflows [13,18,19]. This interpretation is also consistent with the predominance of algorithm or model development and system or tool development across the mapped evidence base [30,40,41].

### Validation Maturity and the Translational Gap

The mapped validation approaches indicate limited translational maturity across emergency domains. Only 13 (7.7%) studies reported external or real-case validation, and only 2 (1.2%) studies reported prospective real-world validation. This pattern suggests that many proposed systems have not yet been evaluated under live operational conditions, which limits confidence in their generalizability and implementation readiness. Representative examples of more advanced validation were uncommon and were largely confined to feasibility, observational, or real-world studies [13,18,19], whereas internal experimental and simulation-based validation remained much more common [33-35].

### Underrepresentation of Emerging and Human-Centered Domains

The persistent underrepresentation of disaster mental health, smart city crisis management, and smart city resilience across scenarios, methodologies, and validation levels suggests that these areas remain largely conceptual [159,160,162,163]. This finding is notable given the increasing recognition of psychological resilience, urban sensing, and human-machine coordination as important components of modern emergency management. Similarly, the limited use of human-in-the-loop evaluation and user-centered validation highlights an ongoing disconnect between technical development and frontline operational realities [38,39].

### Implications for Future Emergency AI Systems

Collectively, these findings suggest that future progress in emergency and disaster AI will depend less on incremental algorithmic refinement alone and more on system-level integration, validation maturity, and deployment within operational environments. Platforms that combine physiological, environmental, and contextual data with decision-support mechanisms spanning prehospital care, hospital operations, and public health coordination may help address the fragmentation and validation limitations identified in this review [13,34,118,120,122].

### Limitations

This review has several limitations. First, the evidence base was heterogeneous in the study design, the terminology, and validation reporting, which limited direct comparability across studies. Second, a substantial proportion of the included literature consisted of conference proceedings and early-stage technical studies, which may report preliminary systems with limited operational validation. Although Google Scholar was searched as a supplementary source, its limited reproducibility and constrained advanced-search functionality may have reduced sensitivity for identifying additional unique gray literature records beyond those captured through bibliographic and engineering databases. Third, only English-language studies were included. Finally, because this was a scoping review, the aim was to map the breadth of the literature rather than to assess pooled effectiveness or establish comparative performance across AI approaches.

### Conclusions

AI research in DM is growing rapidly but remains characterized by thematic concentration, early-stage validation, and limited real-world integration. Progress in the field will likely depend on more integrated systems, stronger external and prospective evaluation, and closer alignment with operational emergency workflows. This scoping review provides a structured overview of the current evidence and highlights priorities for future research and implementation.

## Supplementary material

10.2196/90848 Multimedia Appendix 1 Search strings used for the disaster medicine scoping review.

10.2196/90848 Multimedia Appendix 2 Data extraction sheet for the included articles.

10.2196/90848 Checklist 1 PRISMA-ScR checklist.

## Abbreviations

DM: disaster medicine
PRISMA-ScR: Preferred Reporting Items for Systematic Reviews and Meta-Analyses Extension for Scoping Reviews
