# Supplementary material for: AI in Disaster Medicine: Scoping Review of Methods, Validation, and System Integration
Source: JMIR AI. 2026 Aug 4;5:e90848. doi: 10.2196/90848 (PMC13436798; doi:10.2196/90848)
Supplement: Supplementary file 1 [file ai-v5-e90848-s001.docx]

**(**

**"Disaster Medicine"[Mesh] OR "Disaster Planning"[Mesh] OR "Mass Casualty Incidents"[Mesh]**

**OR "Emergency Preparedness"[Mesh] OR disaster*[tiab] OR "mass casualty"[tiab]**

**OR "mass gathering*"[tiab] OR emergency[tiab]**

**)**

**AND**

**(**

**"Artificial Intelligence"[Mesh] OR "Machine Learning"[Mesh]**

**OR "Clinical Decision Support Systems"[Mesh]**

**OR "deep learning"[tiab] OR "machine learning"[tiab]**

**OR "artificial intelligence"[tiab] OR "decision support"[tiab]**

**OR "predictive model*"[tiab] OR algorithm*[tiab]**

**)**

**AND**

**(**

**"Health Systems"[Mesh] OR hospital*[tiab]**

**OR "emergency medical services"[tiab] OR EMS[tiab]**

**OR "emergency department"[tiab] OR healthcare[tiab]**

**OR "public health"[tiab]**

**)**

TITLE-ABS-KEY(

disaster* OR "disaster medicine" OR "disaster response"

OR "emergency preparedness" OR "mass casualty" OR "mass gathering*"

)

AND TITLE-ABS-KEY(

"artificial intelligence" OR "machine learning" OR "deep learning"

OR "decision support" OR "predictive model*" OR algorithm*

)

AND TITLE-ABS-KEY(

"health system*" OR hospital* OR "emergency medical services"

OR EMS OR "emergency department" OR healthcare OR "public health"

)

("artificial intelligence" OR "machine learning" OR "deep learning" OR algorithm)

AND

(disaster OR "disaster response" OR "emergency preparedness" OR "mass casualty" OR "mass gathering")

AND

(healthcare OR hospital OR "emergency medical services" OR "emergency department" OR "public health")

(disaster OR "disaster medicine" OR "disaster response" OR "emergency preparedness"

OR "mass casualty" OR "mass gathering"):ti,ab,kw

AND

("artificial intelligence" OR "machine learning" OR "deep learning"

OR "decision support" OR "predictive model" OR algorithm):ti,ab,kw

AND

(healthcare OR hospital OR "emergency medical services"

OR "emergency department" OR "public health"):ti,ab,kw

("artificial intelligence" OR "machine learning" OR "deep learning")

AND ("disaster" OR "emergency response" OR "mass casualty" OR "disaster preparedness")

AND ("healthcare" OR "hospital" OR "emergency department")
